# The Lysholm score: Cross cultural validation and evaluation of psychometric properties of the Spanish version

**DOI:** 10.1371/journal.pone.0221376

**Published:** 2019-08-27

**Authors:** Manuel Arroyo-Morales, Jose Martin-Alguacil, Mario Lozano-Lozano, Antonio I. Cuesta-Vargas, Andrés J. Fernández-Fernández, Jose A. González, Yelverton Tegner, Irene Cantarero-Villanueva

**Affiliations:** 1 Department of Physical Therapy, University of Granada, Granada, Spain; 2 Biohealth Research Institute in Granada (ibs.GRANADA), Granada, Spain; 3 Sport and Health University Research Institute (iMUDS), Granada, Spain; 4 Orthopedic Surgery Service Hospital Nuestra Señora de la Salud, Granada, Spain; 5 Department of Physical Therapy, Faculty of Health Sciencies, University of Málaga, Andalucia Tech, Instituto de Investigación en Biomedica de Málaga (IBIMA), Grupo de Clinimetria, Malaga, Spain; 6 School of Clinical Sciences, The Queensland University of Technology, Brisbane, Australia; 7 Department of Computer Science, University of Sheffield, Sheffield, United Kingdom; 8 Division of Medical Sciences, Department of Health Sciences, Luleå University of Technology, Luleå, Sweden; Universidad de Leon, SPAIN

## Abstract

**Background:**

This study aims at assessing the validity and reliability of the Spanish version of the Lysholm score, a widely used instrument for assessing knee function and activity level after ligament injuries.

**Methods:**

Ninety-five participants (67.4% male, 22±5 years) completed the questionnaire twice within 7 days and a subsample of 42 participants completed a test-retest reliability. Reliability, validity and feasibility psychometric properties were studied. The validity of the questionnaire was analysed using ceiling and floor effects. Factor structure and construct validity were analysed with the SF-36, the Hip and Knee Questionnaire (HKQ) and one leg jump test (OLJT).

**Results:**

Criterion validity with the SF-36 Physical State was moderate (r = 0.50 and p<0.01), poor and inverse relationship (r = -0.31, p<0.01) with HKQ and positive moderate (r = 0.59, p<0.01) with OLJT. Measurement error from MDC_90_ was 3.9%. Exploratory factor analysis demonstrated a one-factor solution explaining 51.5% of total variance. The x2 test for the one-factor model was significant (x2 = 29.58, df = 20, p < 0.08). Test-retest reliability level was high (ICC2.1 = 0.92, p<0.01) and also the internal consistency (α = 0.77).

**Conclusion:**

The Spanish Lysholm score demonstrated that it is a reliable and valid instrument that can be used to assess knee function after ligament injuries.

## Introduction

Anterior cruciate ligament (ACL) injuries account for more than 50% of all sustained knee injuries [1]. In fact, ACL rupture has epidemiological importance of the first order, since it has been estimated that annually one out of every 3000 people suffers an ACL tear in the United States. Focusing on Europe, and more specifically on Spain, this percentage reaches 40% [2,3]. Approximately 70% of all ACL injuries are noncontact in nature and 30% are contact injuries [4]. In addition, ACL lesions have been associated with giving-way episodes and development of meniscus tears and knee osteoarthritis [5]. One study states that the incidence of meniscus tears in patients with an ACL lesion is 40% in the first year, 60% in the fifth year and up to 80% 10 years after the injury [6]. It is important to highlight the relationship between the ACL lesion and the stability and activity of the patient. A third of people with an ACL injury will compensate well and will successfully return to their activities without surgery. However, another third could return to recreational activities with effort and a last third may not be able to return due to instability of the knee, requiring surgery [7]. For all this, it is vitally important to monitor patients over time after the ACL injury. In addition, the evaluation of the medium and long-term consequences must be carried out. The Lysholm score was developed to evaluate function and activity after ACL surgery in terms of stability [8] and graded activity [9]. It has also been validated as an instrument administered by the patient to measure symptoms and function in patients with various knee injuries [10–12].

Cultural adaptations of the Lysholm score have been developed previously in many languages and countries [13–18]. Spanish is one of the most widely used languages in the world, and its use is projected to increase in future years. In the United States alone, there has been a 200% increase in the number of Spanish speakers since 1980 [19]. A Spanish version of this score could facilitate the comparison of research results from Spanish speaking patients suffering from knee conditions with data previously reported in the literature [20].

This study aims to report the psychometric properties of the Spanish version of the Lysholm score.

## Materials and methods

### Study design and participants

A psychometric prospective observational study was planned to achieve the objective of the study. First, a double forward and backward translation [21] of the Lysholm score [8] was carried out following COSMIN recommendations [22]. A preliminary study was carried out with 35 patients (14 females, age = 22.6±7.0 years) on a waiting list for ACL surgery to evaluate whether the meaning of the original version was maintained in the Lysholm Spanish version (S1 Lysholm Spanish Version).

In the second stage, an observational prospective assessment of the psychometric properties of the Spanish Lysholm score was carried out with 95 native Spanish speakers who were waiting to undergo surgery for an ACL injury.

Characteristics descriptive of the participants, as well as demographic data for patients are shown in **Table 1**.

**Table 1:** Demographic characteristics of the study population.

*Characteristic*	*Cases* *n (%)*	*Age (years)* *Mean (sd)*
***Study Population***	95 (100%)	21.8 (5.4)
**Male**	64 (67.4%)	21.9 (5.9)
**Female**	31 (32.6%)	21.7 (4.25)
***Civil status*** *Single* *Married* *Divorced*	90 (94.7%) 4 (4.2%) 1 (1.1%)	

### Ethical aspects

The CEIC Hospital Universitario “San Cecilio” ethics committee from Granada approved this study (P16-R13). In addition, all participants were informed, both verbally and in writing about the study, and signed the corresponding written informed consent.

### Procedures: Translation and validation

The Lysholm score was translated into Spanish language considering cultural linguistic adaptations to provide the new version of the questionnaire without language difficulties or other conceptual misunderstandings (see S1 Lysholm Spanish Version).

Validity understood as the degree to which the instrument measure the construct it purposes to measure [22] was assessed using content, construct and criterion validity. To determinate the construct validity and factor structure, maximum likelihood extraction (MLE) with *a priori* extraction requirements was employed. The requirements were established in relation to satisfaction of the following criteria: an eigen value >1.0, screeplot inflection and variance >10% [23]. A single factor structure was the result of the exploratory factor analysis [24]. The fit of the confirmatory factor analysis was acceptable if the comparative and normalized fit indices (CFI and NFI, respectively) were greater than 0.90, with a value of 0.08 deemed the acceptable root mean square error of approximation (RMSEA) [25].

*C*onstruct validity was assessed through the concurrent comparison of Spanish version of the Lysholm score with the following two questionnaires: the SF-36 and HKQ questionnaires, as well as the one leg jump test (OLJT) functional knee test. The SF36, HKQ and OLJT were used to assess discriminate construct, divergent validity and convergent validity, respectively. Poor to moderate positive (SF-36 and OLJT) and negative (HFQ) correlations with Lysholm score were expected. The ceiling and floor effects were analyzed by the percentage of patients with maximum and minimum scores, respectively.

The Spanish version of HKQ has 7 items focusing on pain, function and symptoms. This version has shown adequate reliability [26]. The SF-36 is a well-known instrument with 36 questions using a Likert-type scale, which assesses physical and general mental health. The Spanish version of the SF36 has shown adequate validity [27]. Finally, the vertical jump test was measured using an infrared photocell mat (Ergo-jump Globus, Codogne, Italy). The best performance of three trials was used in the analysis [28].

In this study, several aspects of reliability were assessed, such as test-retest reliability and internal consistency. Test-retest reliability was studied comparing the Spanish Lysholm score values at baseline and one week later through the Intraclass Correlation Coefficient (ICC) Type 2.1 in a random sub-sample of 42 participants. An ICC > 0.70 considered to be acceptable test-retest reliability [29]. Internal consistency of the multi-item questionnaire was assessed by Cronbach’s alpha coefficient [30]. As recommended by Nunnally [31], an expected Cronbach´s alpha coefficient of 0.7 or grater was considered to be adequate to confirm internal consistency.

*Feasibility* was assessed by missing responses as calculated from the total number of responses.

*Sensitivity* was studied through the minimally detectable change (MDC_90_) estimation by following the Stratford approach [32].

### Statistical analysis

A sample size of 95 participants was selected in accordance with to ensure stability of the variance–covariance matrix and required range of 4 to 10 responses for each item [33]. The minimum recommended ratio of ten participants per item was satisfied [34].

Distribution and normality were assessed using the one sample Kolmogorov-Smirnov test. Gender differences were assessed using one-way analysis of variance (ANOVA). To verify that there were no significant differences in sociodemographic and clinical characteristics between the total sample and the subsample, a t-student or chi square was carried out, as appropriate. A Pearson’s r correlation coefficient values between 0.3 and 0.7 (0.3 and −0.7) indicate a moderate positive (negative) linear relationship [35]. SPSS version 22.0 for Mac OS (IBM, Chicago, IL) and LISREL version 8.8 for Windows (SSI Inc., Lincolnwood, USA) were selected as the statistical analysis software for this study [36].

## Results

### Validity

**Fig 1:**
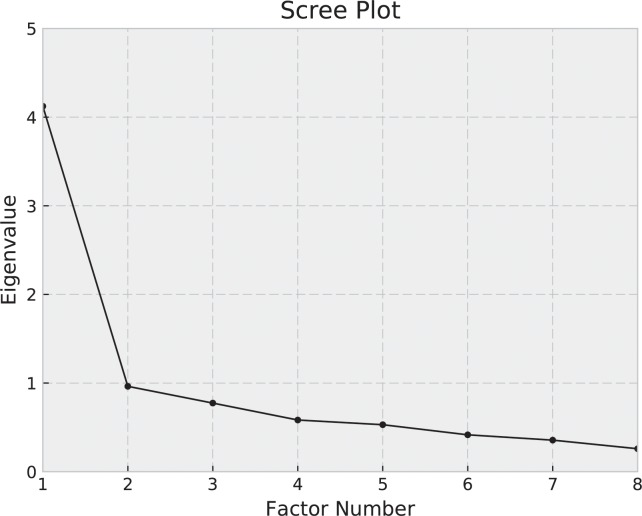
Scree plot of Lysholm score.

A Ceiling effect of 12% and a floor effect of 1% were found in the present study. For construct validity was developed by *factor analysis*, the correlation matrix for the Spanish version of Lysholm was determined to be suitable based on the Kaiser-Meyer-Oklin values (0.86) and Bartlett’s Test of Sphericity (p<0.01). This indicated that the correlation matrix was unlikely to be an identity matrix and, therefore, suitable for MLE. One factor solution was found to be accurate when the *a-priori* criteria were considered, as illustrated in Fig 1.

**Fig 2:**
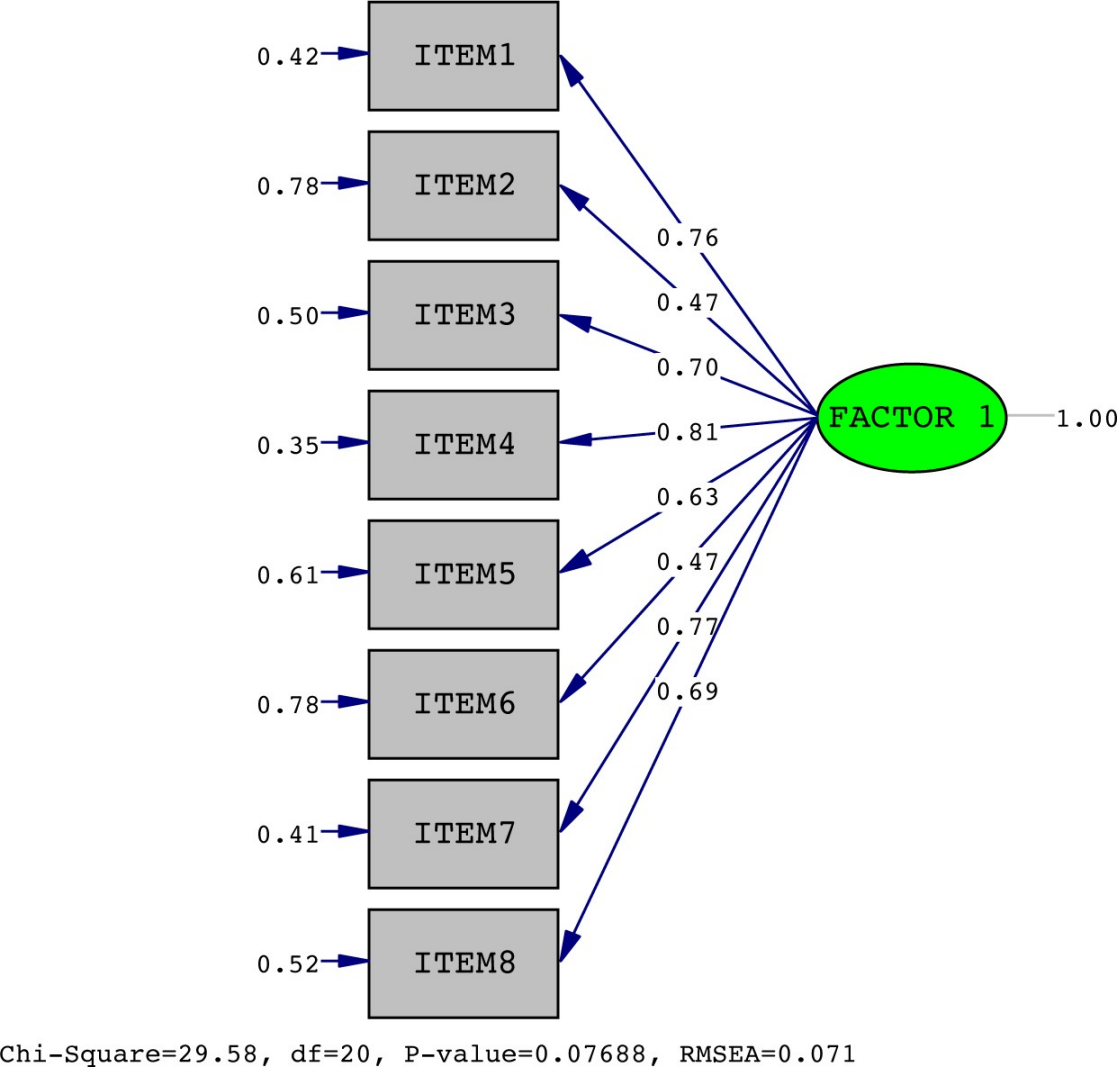
The x^2^ test for the 1-factor model Lysholm score.

Only one of the factors had eigen values >1.0 and accounted for 51.5% of the variance. The item loading for the one-factor solution and the average score for each item are shown in Table 2. The confirmatory factor analysis showed an acceptable fit with a CFI of 0.98 and NFI of 0.94 and appropriate error (RMSEA = 0.07; Standardized RMR = 0.05) under the recommended value of 0.08 [37]. The **x**2 test for the one-factor model was significant (**x**2 = 29.58, df = 20, p < 0.08) (Fig 2).

**Table 2:** Factor loading items for the one-factor solution.

Factor Matrix	
	Component 1
Pain	0.76
Swelling	0.47
Limp	0.70
Squatting	0.81
Instability	0.62
Support	0.47
Stairs climbing	0.77
Locking	0.70

The convergent criterion validity was tested with correlation between the SF-36 Physical Functioning Scale and the Lysholm score and it was moderate and positive (r = -0.30 and p<0.01). There was no significant correlation between the SF-36 Mental Health Subscale and the Lysholm score (r = 0.38; p = 0.09). The correlation between the HFQ and the Lysholm score was moderate and positive (r = 0.50 and p<0.01). Finally, the correlation between OLJT and Lysholm score was moderate and positive (r = 0.59, p<0.01).

### Reliability

Test-retest reliability was high (ICC_2.1_ = 0.92, 95% CI 0.88 to 0.94, p≤0.001). Moreover, there was an appropriate degree of *internal consistency*: α = 0.77 (95% CI, 0.69–0.83). Finally, there were no significant differences between total sample and the subsample in clinical and sociodemographic characteristics.

### Feasibility

For the Lysholm score, there were no significant missing responses. No significant gender differences were found in the item responses.

### Sensitivity

The measurement error from MDC_90_ was 3.9%.

## Discussion

The Spanish version of the Lysholm score showed adequate psychometric properties in patients with ACL injuries when assessed for reliability, validity, feasibility and sensitivity.

In the absence of a real gold standard, the Lysholm score is considered to be the standard for assessing ACL injury deficits [38]. With regard to criterion validity the correlations were higher [22,25] and similar to other validated versions [37]. An unexpected result of this study was the higher correlation between the Lysholm score and the functional jump test which could be interpreted as a reflection of a close relationship between this scale and knee performance in patients with ACL injuries. Feasibility was adequate in this study as there were no missing data and an acceptable error for MDC_90_.

Further affirming the level of validity of the Spanish version of the Lysholm score, acceptable level of ceiling and floor effects were found and higher than those reported in previous studies [37,39], which may be due to the peculiarity of the sample selected in this study (age and type of sport activity of the sample). With respect to factor analysis, this study revealed a satisfactory percentage of total variance explained by one factor at 51.5% which guaranteed a correct confirmatory factor analysis. This one factor structure is in line with the original version [9].

It demonstrated the same level of test-retest reliability than a previous study [12] and a higher level of test-retest reliability than other cross-cultural validation studies [23,39] or a similar ACL injury population [37]. The degree of *internal consistency* (α = 0.77) was lower than in other studies [13], but adequate for health-related studies with patients [22].

This research was not free of limitations. A number of different validations aspects were not assessed in this study, including responsiveness and content validity. Also, only one center was involved. New research is warranted to fill theses gaps of knowledge.

In conclusion, the Spanish version of the Lysholm score has similar reliability and validity to the original version and other adaptations.

## Supporting information

S1 Lysholm Spanish Version Spanish version of Lysholm Scale. (PDF)

## References

[1] Arna Risberg M, Lewek M, Snyder-Mackler L. A systematic review of evidence for anterior cruciate ligament rehabilitation: how much and what type? Phys Ther Sport. 2004;5: 125–145. 10.1016/J.PTSP.2004.02.003

[2] Herrero H, Salinero JJ, Del Coso J. Injuries Among Spanish Male Amateur Soccer Players. Am J Sports Med. 2014;42: 78–85. 10.1177/0363546513507767 24136859

[3] Del Coso J, Herrero H, Salinero JJ. Injuries in Spanish female soccer players. J Sport Heal Sci. 2018;7: 183–190. 10.1016/J.JSHS.2016.09.002 30356460 PMC6180559

[4] Hewett TE, Ford KR, Myer GD. Anterior Cruciate Ligament Injuries in Female Athletes. Am J Sports Med. 2006;34: 490–498. 10.1177/0363546505282619 16382007

[5] Beynnon BD, Johnson RJ, Abate JA, Fleming BC, Nichols CE. Treatment of Anterior Cruciate Ligament Injuries, Part I. Am J Sports Med. 2005;33: 1579–1602. 10.1177/0363546505279913 16199611

[6] Levy AS, Wetzler MJ, Lewars M, Laughlin W. Knee Injuries in Women Collegiate Rugby Players. Am J Sports Med. 1997;25: 360–362. 10.1177/036354659702500315 9167817

[7] Logerstedt DS, Snyder-Mackler L, Ritter RC, Axe MJ, Godges JJ. Knee Stability and Movement Coordination Impairments: Knee Ligament Sprain: Clinical Practice Guidelines Linked to the International Classification of Functioning, Disability, and Health from the Orthopaedic Section of the American Physical Therapy Association. J Orthop Sports Phys Ther. 2010;40: A1 10.2519/JOSPT.2010.0303 20357420 PMC3158982

[8] Tegner Y, Lysholm J. Rating systems in the evaluation of knee ligament injuries. Clin Orthop Relat Res. 1985; 43–9. Available: http://www.ncbi.nlm.nih.gov/pubmed/4028566 4028566

[9] Caplan N, Kader DF. Rating Systems in the Evaluation of Knee Ligament Injuries. Classic Papers in Orthopaedics. London: Springer London; 2014 pp. 201–203. 10.1007/978-1-4471-5451-8_49

[10] Kocher MS, Steadman RJ, Briggs KK, Sterett WI, Hawkins RJ. Reliability, Validity, and Responsiveness of the Lysholm Knee Scale for Various Chondral Disorders of the Knee. J Bone Jt Surgery-American Vol. 2004;86A: 1139–1145. 10.2106/00004623-200406000-00004 15173285

[11] Briggs KK, Kocher MS, Rodkey WG, Steadman JR. Reliability, Validity, and Responsiveness of the Lysholm Knee Score and Tegner Activity Scale for Patients with Meniscal Injury of the Knee. J Bone Jt Surg. 2006;88: 698–705. 10.2106/JBJS.E.00339 16595458

[12] Paxton EW, Fithian DC, Lou Stone M, Silva P. The Reliability and Validity of Knee-Specific and General Health Instruments in Assessing Acute Patellar Dislocation Outcomes. Am J Sports Med. 2003;31: 487–492. 10.1177/03635465030310040201 12860533

[13] Swanenburg J, Koch P, Meier N, Wirth B. Function and activity in patients with knee arthroplasty: validity and reliability of a German version of the Lysholm Score and the Tegner Activity Scale. Swiss Med Wkly. 2014;144: w13976 10.4414/smw.2014.13976 24921654

[14] Piontek T, Ciemniewska-Gorzela K, Naczk J, Cichy K, Szulc A. Linguistic and cultural adaptation into Polish of the IKDC 2000 subjective knee evaluation form and the Lysholm scale. Polish Orthop Traumatol. 2012;77: 115–9. Available: http://www.ncbi.nlm.nih.gov/pubmed/23306298 23306298

[15] Celik D, Coşkunsu D, Kılıçoğlu Ö. Translation and Cultural Adaptation of the Turkish Lysholm Knee Scale: Ease of Use, Validity, and Reliability. Clin Orthop Relat Res. 2013;471: 2602–2610. 10.1007/s11999-013-3046-z 23666590 PMC3705057

[16] Cerciello S, Corona K, Morris BJ, Visonà E, Maccauro G, Maffulli N, et al Cross-cultural adaptation and validation of the Italian versions of the Kujala, Larsen, Lysholm and Fulkerson scores in patients with patellofemoral disorders. J Orthop Traumatol. 2018;19: 18 10.1186/s10195-018-0508-9 30209631 PMC6135726

[17] Wang W, Liu L, Chang X, Jia ZY, Zhao JZ, Xu WD. Cross-cultural translation of the Lysholm knee score in Chinese and its validation in patients with anterior cruciate ligament injury. BMC Musculoskelet Disord. 2016;17: 436 10.1186/s12891-016-1283-5 27756266 PMC5069932

[18] Eshuis R, Lentjes GW, Tegner Y, Wolterbeek N, Veen MR. Dutch Translation and Cross-cultural Adaptation of the Lysholm Score and Tegner Activity Scale for Patients With Anterior Cruciate Ligament Injuries. J Orthop Sport Phys Ther. 2016;46: 976–983. 10.2519/jospt.2016.6566 27681449

[19] Bureau UC. Language Use in the United States: 2011. Available: https://www.census.gov/library/publications/2013/acs/acs-22.html

[20] Flores G. Language Barriers to Health Care in the United States. N Engl J Med. 2006;355: 229–231. 10.1056/NEJMp058316 16855260

[21] Beaton DE, Bombardier C, Guillemin F, Ferraz MB. Guidelines for the process of cross-cultural adaptation of self-report measures. Spine (Phila Pa 1976). 2000;25: 3186–91. Available: http://www.ncbi.nlm.nih.gov/pubmed/11124735 11124735 10.1097/00007632-200012150-00014

[22] Mokkink LB, Terwee CB, Patrick DL, Alonso J, Stratford PW, Knol DL, et al The COSMIN study reached international consensus on taxonomy, terminology, and definitions of measurement properties for health-related patient-reported outcomes. J Clin Epidemiol. 2010;63: 737–745. 10.1016/j.jclinepi.2010.02.006 20494804

[23] Muñiz J, Elosua P, Hambleton RK. Directrices para la traducción y adaptación de los tests: Segunda edición. Psicothema. 2013;25: 151–157. 10.7334/psicothema2013.24 23628527

[24] Field A. Discovering Statistics using IBM SPSS Statistics. Sage; 2013.

[25] Hu L, Bentler PM. Cutoff criteria for fit indexes in covariance structure analysis: Conventional criteria versus new alternatives. Struct Equ Model A Multidiscip J. 1999;6: 1–55. 10.1080/10705519909540118

[26] Castellet E, Ares O, Celaya F, Valentí-Azcárate A, Salvador A, Torres A, et al Transcultural adaptation and validation of the “Hip and Knee” questionnaire into Spanish. Health Qual Life Outcomes. 2014;12: 76 10.1186/1477-7525-12-76 24885248 PMC4035858

[27] Alonso J, Prieto L, Antó JM. [The Spanish version of the SF-36 Health Survey (the SF-36 health questionnaire): an instrument for measuring clinical results]. Med Clin (Barc). 1995;104: 771–6. Available: http://www.ncbi.nlm.nih.gov/pubmed/7783470 7783470

[28] Bosco C, Mognoni P, Luhtanen P. Relationship between isokinetic performance and ballistic movement. Eur J Appl Physiol Occup Physiol. 1983;51: 357–64. Available: http://www.ncbi.nlm.nih.gov/pubmed/6685034 6685034 10.1007/BF00429072

[29] Machin D, Fayers P. Quality of life: the assessment, analysis and interpretation of patient-reported outcomes [Internet]. Wiley; 2013 Available: https://www.wiley.com/en-us/Quality+of+Life%3A+The+Assessment%2C+Analysis+and+Interpretation+of+Patient+reported+Outcomes%2C+2nd+Edition-p-9781118699454

[30] Cronbach LJ. Coefficient alpha and the internal structure of tests. Psychometrika. 1951;16: 297–334. 10.1007/BF02310555

[31] Nunnally JC. Psychometric theory [Internet]. McGraw-Hill; 1978 Available: https://books.google.es/books/about/Psychometric_theory.html?id=WE59AAAAMAAJ&redir_esc=y

[32] Stratford PW. Getting More from the Literature: Estimating the Standard Error of Measurement from Reliability Studies. Physiotherapy. 2004;56: 27–30. 10.2310/6640.2004.15377

[33] Terwee CB, Bot SDM, de Boer MR, van der Windt DAWM, Knol DL, Dekker J, et al Quality criteria were proposed for measurement properties of health status questionnaires. J Clin Epidemiol. 2007;60: 34–42. 10.1016/j.jclinepi.2006.03.012 17161752

[34] Mundfrom DJ, Shaw DG, Ke TL. Minimum Sample Size Recommendations for Conducting Factor Analyses. Int J Test. 2005;5: 159–168. 10.1207/s15327574ijt0502_4

[35] Ratner B. The correlation coefficient: Its values range between +1/−1, or do they? J Targeting, Meas Anal Mark. 2009;17: 139–142. 10.1057/jt.2009.5

[36] Martínez-González MA, Sánchez-Villegas A, Toledo Atucha E, Faulín FJ. Bioestadística amigable. Elsevier; 2014.

[37] Briggs KK, Lysholm J, Tegner Y, Rodkey WG, Kocher MS, Steadman JR. The Reliability, Validity, and Responsiveness of the Lysholm Score and Tegner Activity Scale for Anterior Cruciate Ligament Injuries of the Knee. Am J Sports Med. 2009;37: 890–897. 10.1177/0363546508330143 19261899

[38] Johnson DS, Smith RB. Outcome measurement in the ACL deficient knee—what’s the score? Knee. 2001;8: 51–7. Available: http://www.ncbi.nlm.nih.gov/pubmed/11248569 11248569 10.1016/s0968-0160(01)00068-0

[39] Briggs KK, Steadman JR, Hay CJ, Hines SL. Lysholm Score and Tegner Activity Level in Individuals with Normal Knees. Am J Sports Med. 2009;37: 898–901. 10.1177/0363546508330149 19307332

