# Supplementary material for: The Lysholm score: Cross cultural validation and evaluation of psychometric properties of the Spanish version
Source: PLoS One. 2019 Aug 27;14(8):e0221376. doi: 10.1371/journal.pone.0221376 (PMC6711518; doi:10.1371/journal.pone.0221376)
Supplement: S1 Lysholm Spanish Version — (PDF) [file pone.0221376.s001.pdf]

ESCALA LYSHOLM

Este cuestionario ha sido diseñado para dar información a su terapeuta acerca de cómo el dolor de rodilla ha afectado su capacidad para desenvolverse en su vida cotidiana. Por favor, conteste a todas las preguntas indicando la casilla que mejor describa su condición actual. Las diferentes secciones del cuestionario se refieren al estado de su rodilla en las últimas 4 semanas. Por favor en cada ítem seleccione **sólo una** de las

1. COJERA

- ☐ Ninguna
- ☐ Leve o periódica
- ☐ Grave y constante

2. APOYO

- ☐ Ninguno
- ☐ Bastón o muleta
- ☐ No puede apoyar la pierna

3. DOLOR

- ☐ Ninguno
- ☐ Inconstante y leve al realizar esfuerzos intensos
- ☐ Marcado durante al realizar esfuerzos intensos
- ☐ Marcado durante o tras caminar más de 2 km
- ☐ Marcado durante o tras caminar menos de 2 km
- ☐ Dolor constante

4. INESTABILIDAD

- ☐ Nunca
- ☐ Raramente al practicar actividades deportivas u otros esfuerzos intensos
- ☐ Con frecuencia al practicar actividades deportivas u otros esfuerzos intensos
- ☐ De vez en cuando en las tareas cotidianas
- ☐ A menudo en las tareas cotidianas
- ☐ En cada paso

5. BLOQUEO

- ☐ Sin bloqueo y si sensación de roce
- ☐ Sensación de roce a cada paso
- ☐ Bloqueo ocasional
- ☐ Bloqueo frecuente
- ☐ Bloqueo durante la valoración

6. INFLAMACIÓN

- ☐ Ninguna
- ☐ En esfuerzos intentos
- ☐ En esfuerzos cotidianos
- ☐ Constante

7. SUBIR ESCALERAS

- ☐ Sin problemas
- ☐ Problemas leves
- ☐ Sube de los escalones de uno en uno
- ☐ Imposible

8. CUCLILLAS

- ☐ Sin problemas
- ☐ Problemas leves
- ☐ No más de 90 grados
- ☐ Imposible

¡GRACIAS POR SU PARTICIPACIÓN!
